# The strigolactone biosynthesis gene *DWARF27* is co-opted in rhizobium symbiosis

**DOI:** 10.1186/s12870-015-0651-x

**Published:** 2015-10-26

**Authors:** Arjan van Zeijl, Wei Liu, Ting Ting Xiao, Wouter Kohlen, Wei-Cai Yang, Ton Bisseling, René Geurts

**Affiliations:** Department of Plant Science, Laboratory of Molecular Biology, Wageningen University, Droevendaalsesteeg 1, 6708 PB Wageningen, The Netherlands; State Key Laboratory of Molecular and Developmental Biology, Institute of Genetics and Developmental Biology, Chinese Academy of Sciences, Beijing, 100101 China

**Keywords:** DWARF27, CCD7, CCD8, *Medicago truncatula*, Nodulation, Lipo-chitooligosaccharide, Nod factors, Rhizobium, Phosphate starvation, Strigolactones

## Abstract

**Background:**

Strigolactones are a class of plant hormones whose biosynthesis is activated in response to phosphate starvation. This involves several enzymes, including the carotenoid cleavage dioxygenases 7 (CCD7) and CCD8 and the carotenoid isomerase DWARF27 (D27). *D27* expression is known to be responsive to phosphate starvation. In *Medicago truncatula* and rice (*Oryza sativa*) this transcriptional response requires the GRAS-type proteins NSP1 and NSP2; both proteins are essential for rhizobium induced root nodule formation in legumes. In line with this, we questioned whether MtNSP1-MtNSP2 dependent *MtD27* regulation is co-opted in rhizobium symbiosis.

**Results:**

We provide evidence that MtD27 is involved in strigolactone biosynthesis in *M. truncatula* roots upon phosphate stress. Spatiotemporal expression studies revealed that this gene is also highly expressed in nodule primordia and subsequently becomes restricted to the meristem and distal infection zone of a mature nodules. A similar expression pattern was found for *MtCCD7* and *MtCCD8.* Rhizobium lipo-chitooligosaccharide (LCO) application experiments revealed that of these genes *MtD27* is most responsive in an MtNSP1 and MtNSP2 dependent manner. Symbiotic expression of *MtD27* requires components of the symbiosis signaling pathway; including MtDMI1, MtDMI2, MtDMI3/MtCCaMK and in part MtERN1. This in contrast to *MtD27* expression upon phosphate starvation, which only requires MtNSP1 and MtNSP2.

**Conclusion:**

Our data show that the phosphate-starvation responsive strigolactone biosynthesis gene *MtD27* is also rapidly induced by rhizobium LCO signals in an MtNSP1 and MtNSP2-dependent manner. Additionally, we show that *MtD27* is co-expressed with *MtCCD7* and *MtCCD8* in nodule primordia and in the infection zone of mature nodules.

**Electronic supplementary material:**

The online version of this article (doi:10.1186/s12870-015-0651-x) contains supplementary material, which is available to authorized users.

## Background

Legumes evolved the capacity to live in an intimate endosymbiosis with nitrogen-fixing rhizobium bacteria. To host rhizobia intracellularly, nodules are formed on the root of the plant. These nodules provide optimal physiological conditions to the bacteria to fix atmospheric nitrogen gas into ammonia. Recent studies have suggested a role for strigolactones in rhizobium symbiosis [1–6]. Here we focus on expression of the strigolactone biosynthesis gene *DWARF27* during the legume-rhizobium interaction.

Nodule formation is initiated upon perception of lipo-chitooligosaccharide (LCO) signals excreted by compatible rhizobium bacteria [7]. These signals mitotically activate root cortical and pericycle cells, resulting in the formation of a nodule primordium [7, 8]. Rhizobium LCOs (also known as Nod factors) are also required to initiate an infection process to establish intracellular accommodation of the prokaryotic endosymbiont. This infection process starts in curled root hairs where a tube-like structure is formed intracellularly, which guides the rhizobia to the newly formed nodule primordium. There, the rhizobia are released as organelle-like structures (named symbiosomes), which remain surrounded by a plant-derived membrane. These symbiosomes act as nitrogen-fixing units that provide ammonia to the plant cell in exchange of nutrients [7].

Rhizobium LCO signals are perceived by a specific set of LysM-type receptor kinases at the root epidermis. This activates a signaling cascade that is shared with a more ancient endosymbiosis; that between land plants and arbuscular mycorrhizal fungi [7]. This signaling cascade consists of a plasma membrane localized LRR-type receptor kinase (named MtDMI2 in *Medicago truncatula*), a cation ion channel in the nuclear envelope (MtDMI1 in *M. truncatula*), and a nuclear localized Ca^2+^/calmodulin dependent protein kinase (MtCCaMK/MtDMI3 in *M. truncatula*) [9–11]. Downstream of this cascade, CCaMK phosphorylates the transcriptional activator CYCLOPS, which orchestrates symbiotic root nodule development in conjunction with other transcription factors [12]. Among these are the GRAS-type transcriptional regulators NSP1 and NSP2 [13–15]. Experiments in heterologous systems have revealed that NSP2 can form a heterodimer with NSP1, suggesting a regulatory link between both proteins [16]. Both transcriptional regulators are essential for rhizobium symbiosis, but also promote mycorrhizal infection [17–20].

Under non-symbiotic conditions, NSP1 and NSP2 regulate the expression of *DWARF27* (*D27*), which encodes a key enzyme in strigolactone biosynthesis [17, 21]. Strigolactones are a class of plant hormones derived from all-trans-β-carotene [22–24]. They are produced mainly in the root of the plant and the biosynthesis of their basic structure involves at least three plastid-localized enzymes; the carotenoid isomerase D27 and the carotenoid cleavage dioxygenases 7 (CCD7) and CCD8 [22]. The subsequent activity of these three enzymes results in biosynthesis of carlactone, a precursor of strigolactones. The conversion of carlactone to strigolactones is not completely resolved yet, but there is strong evidence to support that this involves a cytochrome P450 enzyme, encoded by *MAX1* in Arabidopsis (*Arabidopsis thaliana*) and rice (*Oryza sativa*) [25–28]. This biosynthetic pathway is under the control of a nutrient sensing mechanism and/or the nutrient status of the plant [29–31]. Especially in plants grown under phosphate-limited conditions, *D27* expression and subsequent strigolactone production is markedly increased in an NSP1/NSP2-dependent manner [17, 29].

Under phosphate limitation, strigolactones are exuded into the rhizosphere to attract arbuscular mycorrhizal fungi [31–34]. These obligatory biotrophic (symbiotic) fungi can sense strigolactones and respond with an increased hyphal branching, thereby, promoting colonization of plant roots [32, 35, 36]. The endomycorrhizal symbiosis facilitates nutrient exchange between fungus and plant. Mycelium that remains in the soil markedly increases the plant root capacity to access nutrients, especially immobile phosphates. The plant receives these nutrients from the fungi at the expense of carbohydrates [37]. Although strigolactones are not essential for establishment of an endomycorrhizal symbiosis, they contribute significantly to increasing root infection levels [33].

Several reports suggest also a role for strigolactones in legume nodule formation. In *Medicago sativa* and *M. truncatula*, application of 0.1 μM GR24 was shown to promote nodule formation, whereas slightly higher concentrations inhibited nodule formation in *M. truncatula* [1, 5]. In pea (*Pisum sativum*) and *Lotus japonicus*, strigolactone deficient mutants were shown to produce less nodules, which could be rescued by external application of GR24 [2–4]. Furthermore, in *M. truncatula* root hairs *MtD27* and *MtCCD8* expression is increased at 5 days post rhizobium inoculation [6]. This implies that *MtD27* expression is regulated also in a symbiotic context. However, the precise regulatory network remains unknown.

Here, we show that in *M. truncatula MtD27* expression is induced by rhizobium LCOs in an MtNSP1 and MtNSP2-dependent manner, similar as found for the induction of *MtD27* by phosphate starvation. However, only induction of *MtD27* by rhizobium LCOs requires the symbiosis signaling cascade. Using promoter-reporter constructs, we show that *MtD27* is expressed throughout nodule formation. After early activation in the epidermis, its expression becomes restricted to the nodule primordium and subsequently to the nodule meristem and infection zone. Furthermore, we show that in nodule primordia and mature nodules *MtD27* is co-expressed with *MtCCD7* and *MtCCD8*.

## Results

### MtD27 is involved in strigolactone biosynthesis

**Fig. 1:**
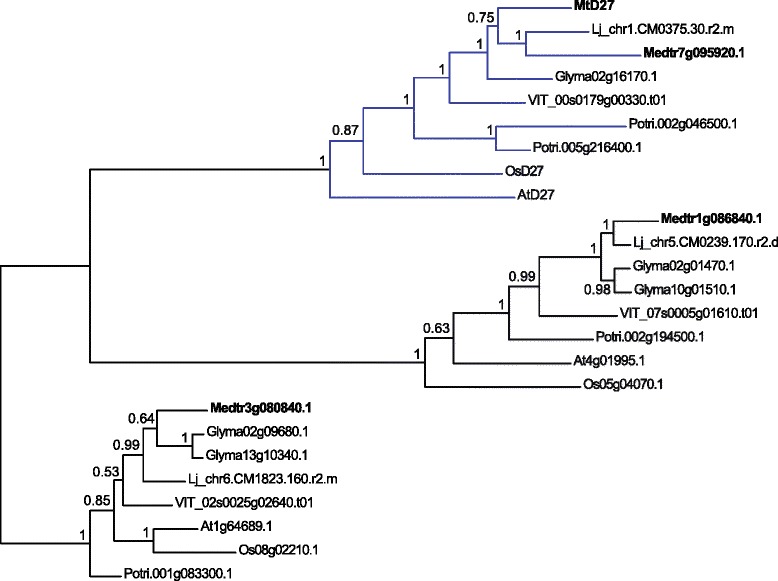
Bayesian phylogeny of D27 and D27-like proteins. Phylogeny was reconstructed based on an alignment of D27 and D27-like proteins from Arabidopsis (At), soybean (*Glycine max*) (Glyma), *Lotus japonicus* (Lj), *M. truncatula* (Medtr), rice (Os), poplar (*Populus trichocarpa*) (Potri) and grapevine (*Vitis vinifera*) (VIT). Branch support is indicated by posterior probabilities. Terminals are labeled by their gene name or genbank identifier. Proteins identified from *M. truncatula* are highlighted in bold. The D27 orthology group containing rice OsD27 (gi|2549466546) and Arabidopsis AtD27 (At1G03055) is highlighted in blue. Mid-point rooting was applied for better tree visualization

The *M. truncatula D27* ortholog (Medtr1g471050) was identified previously [17]. By searching the *M. truncatula* genome annotation Mt4.0, we noted the presence of 3 close homologs of the original *MtD27* gene. To get insight in the relation of these genes to *MtD27*, we conducted a phylogenetic analysis based on an alignment of D27(-like) proteins of different plant species, including rice and Arabidopsis (Fig. 1). This showed that these sequences grouped in three separate phylogenetic clades (Fig. 1), consistent with a previous report [38]. Interestingly, the D27 clade contains 2 proteins from *M. truncatula*: MtD27 [17] as well as a close homolog Medtr7g095920. This shows that *M. truncatula* contains 2 putative *D27* genes that could function redundantly. Expression of *MtD27* and subsequent strigolactone production is known to be induced by phosphate starvation [17]. To get first insight whether Medtr7g095920 may also have a function in strigolactone production in *M. truncatula* roots, we examined its expression pattern. Analysis of publically available microarray data showed that Medtr7g095920 expression is relatively low, and has limited overlap with expression of *MtD27* [39]. In roots, Medtr7g095920 expression is not induced by phosphate starvation. This in contrast to *MtD27* (Additional file 1) [17]. This suggests that under phosphate limiting conditions only *MtD27* might be involved in strigolactone biosynthesis in *M. truncatula* roots.

**Fig. 2:**
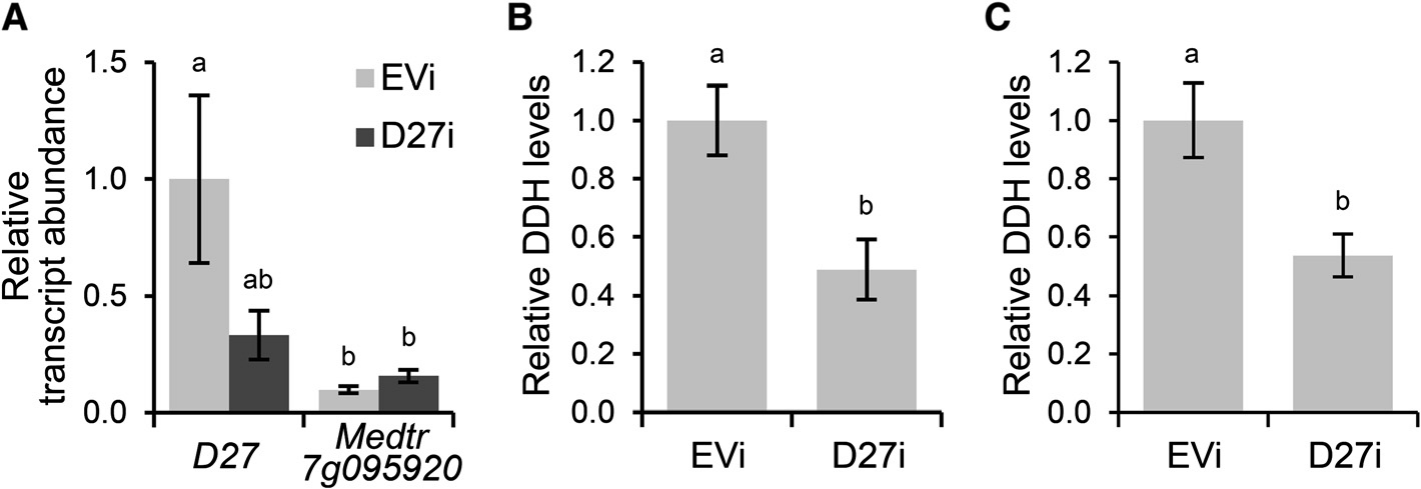
Effect of *MtD27* knock-down on strigolactone biosynthesis. **a** Relative transcript abundance as determined by qRT-PCR of *MtD27* and Medtr7g095920 in *M. truncatula* transgenic roots expressing an empty vector control construct (EVi) or *MtD27* RNAi construct (D27i). Relative transcript abundance was normalized against *MtD27* transcript abundance in roots transformed with the empty vector control (EVi). **b** Relative quantity of the strigolactone didehydro-orobanchol (DDH) (peak area/g FW) in root exudates collected from *M. truncatula* transgenic roots expressing an empty vector control construct (EVi) or *MtD27* RNAi construct (D27i). **c** Relative quantity of the strigolactone didehydro-orobanchol (DDH) (peak area/g FW) in root extracts collected from *M. truncatula* transgenic roots expressing an empty vector control construct (EVi) or *MtD27* RNAi construct (D27i). Data shown represent means of 4-5 biological replicates ± SEM. Different letters above bars indicate statistical difference (*p* < 0.05, students’ *t*-test)

Next, we determined whether MtD27 represents a functional enzyme in the strigolactone biosynthesis pathway. To this end, we generated *M. truncatula* compound plants bearing transgenic roots in which *MtD27* expression was reduced through RNAi-mediated knock-down. This reduced *MtD27* expression by ~65 %, though with substantial variation (Fig. 2a). In contrast, expression of Medtr7g095920 was not reduced, but lower than *MtD27* (Fig. 2a). Measurements of root extracts as well as root exudates collected from the *MtD27* RNAi roots showed a 45-55 % reduction in strigolactone concentrations compared to that in the empty vector control (Fig. 2b and c). Taken together, this shows that MtD27 is involved in strigolactone biosynthesis and represents a functional ortholog of OsD27 from rice.

### Expression of *MtD27* is increased upon perception of rhizobium LCOs in an MtNSP1 and MtNSP2-dependent manner

**Fig. 3:**
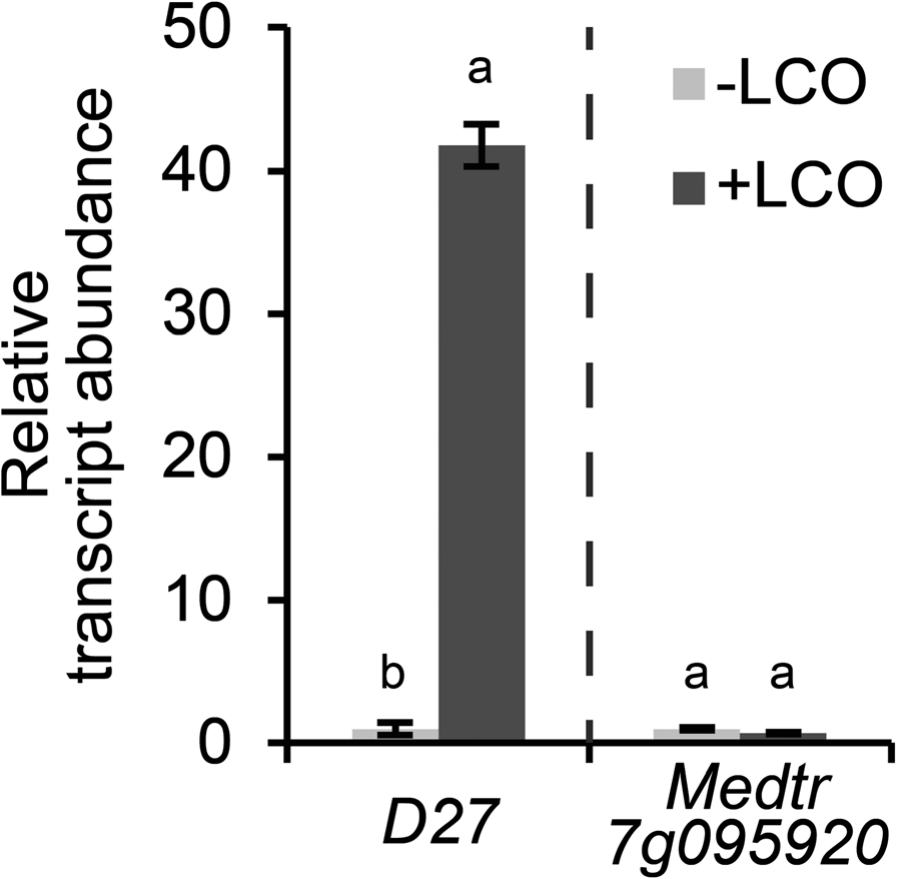
Relative transcript abundance of *MtD27* and Medtr7g095920 after application of rhizobium LCOs. Relative transcript abundance as determined by qRT-PCR of *MtD27* and Medtr7g095920 in *M. truncatula* root susceptible zones 3 h after mock (-LCO) or *S. meliloti* LCO (10^-9^ M) (+LCO) treatment. Data shown represent means of 2 biological replicates that each were analyzed in 3-fold (technical replicates) ± SEM. For each gene, transcript abundance was normalized against that of the mock-treated wild type. Different letters above bars indicate statistical difference (*p* < 0.05, students’ *t*-test)

To obtain insight in the symbiotic function of *MtD27*, we first determined whether its expression is responsive to rhizobium LCOs. Quantitative RT-PCR (qRT-PCR) reactions on RNA isolated from *M. truncatula* roots treated with *Sinorhizobium meliloti* LCOs (~10^-9^ M) for 3 h revealed that expression of *MtD27* is strongly induced (Fig. 3). To determine whether Medtr7g095920 is also responsive to rhizobium LCOs, we quantified its expression as well. This showed that, unlike *MtD27*, Medtr7g095920 is not responsive (Fig. 3). The low expression of Medtr7g095920 in combination with its non-responsiveness to phosphate starvation and rhizobium LCOs let us to decide to focus further studies on the symbiotic function of *MtD27*.

**Fig. 4:**
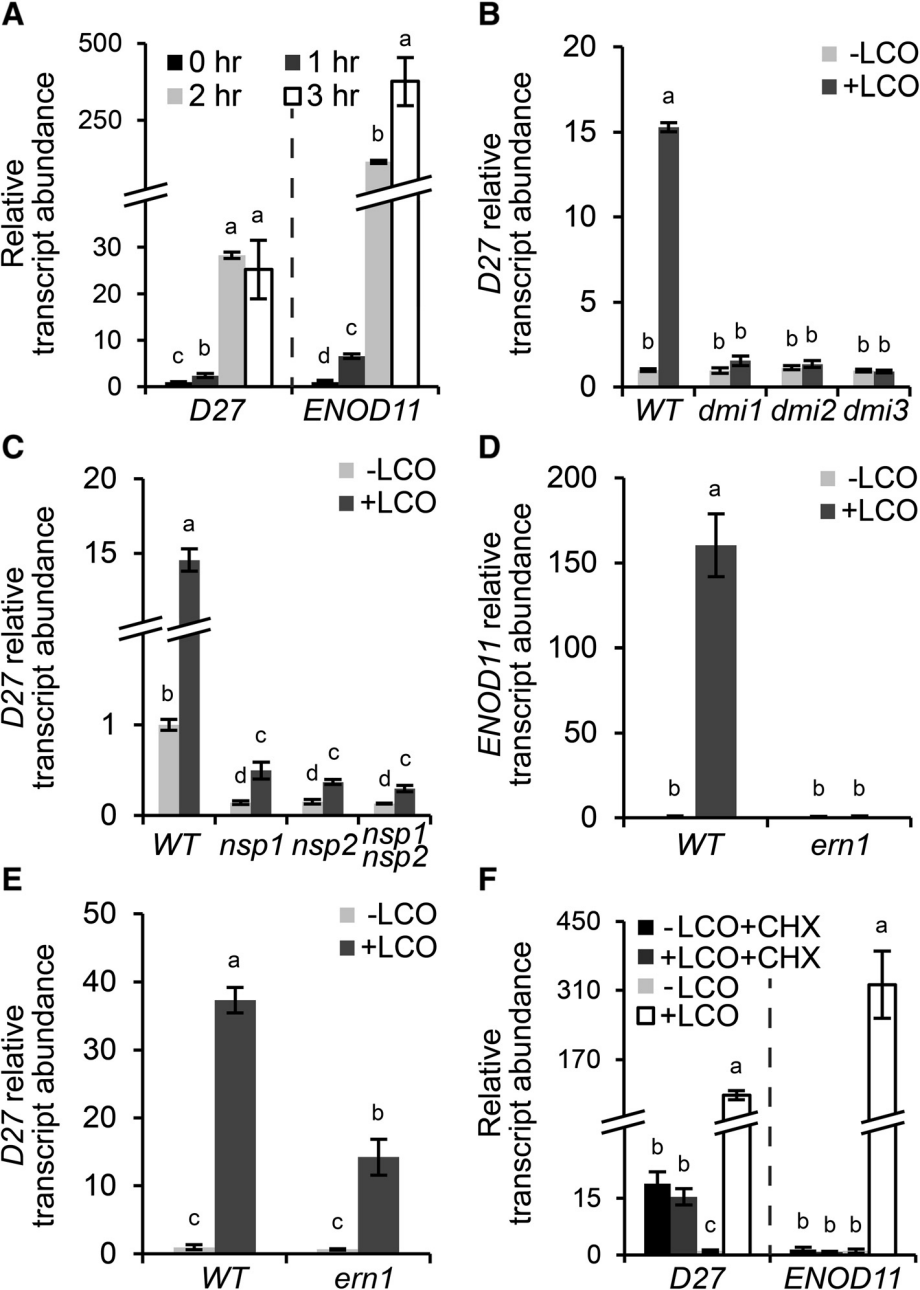
Symbiotic *MtD27* expression is under direct control of *S. meliloti* LCO signaling. **a** Relative transcript abundance as determined by qRT-PCR of *MtD27* and *MtENOD11* in *M. truncatula* root susceptible zones after 0, 1, 2 or 3 h of LCO treatment (10^-9^ M). **b** Relative transcript abundance as determined by qRT-PCR of *MtD27* at 3 h after mock (-LCO) or rhizobium LCO (10^-9^ M) (+LCO) treatment in wild type, *Mtdmi1*, *Mtdmi2* and *Mtdmi3*. **c** Relative transcript abundance as determined by qRT-PCR of *MtD27* at 3 h after mock (-LCO) or rhizobium LCO (10^-9^ M) (+LCO) treatment in wild type, *Mtnsp1*, *Mtnsp2* and *Mtnsp1 Mtnsp2*. **d** Relative transcript abundance as determined by qRT-PCR of *MtENOD11* at 3 h after mock (-LCO) or rhizobium LCO (10^-9^ M) (+LCO) treatment in wild type and *Mtern1*. **e** Relative transcript abundance as determined by qRT-PCR of *MtD27* at 3 h after mock (-LCO) or rhizobium LCO (10^-9^ M) (+LCO) treatment in wild type and *Mtern1*. **f** Relative transcript abundance as determined by qRT-PCR of *MtD27* and *MtENOD11* after mock (-LCO) or rhizobium LCO (10^-9^ M) (+LCO) treatment, in presence or absence of 50 μM cycloheximide (CHX). Data shown represent means of 3 biological replicates that each were analyzed in 3-fold (technical replicates) ± SEM. For each gene, transcript abundance was normalized against that of the mock-treated wild type. Different letters above bars indicate statistical difference (*p* < 0.05, students’ *t*-test)

To acquire more insight in the symbiotic responsiveness of *MtD27*, we conducted a time series qRT-PCR experiment. To this end, *M. truncatula* seedlings were grown in Fåhraeus slides [40], a system optimized to study early responses induced by rhizobium LCOs in the root epidermis. *M. truncatula* roots of wild-type plants were treated with *S. meliloti* LCOs (~10^-9^ M) for 0, 1, 2 and 3 h. Subsequently, total RNA was isolated from the so-called susceptible zone, a region of about 1 cm just above the root meristem. Expression analysis by qRT-PCR showed a slight induction of *MtD27* already at 1 h post LCO application, and a strong >20-fold induction after 2-3 h compared to mock-treated roots (Fig. 4a). This timing and induction level is comparable to that of *MtENOD11*; a gene frequently used as marker for rhizobium LCO-induced signaling in *M. truncatula* [16, 41] (Fig. 4a)*.*

Induction of *MtENOD11* expression by rhizobium LCOs requires a signaling module downstream of LCO perception consisting of MtDMI1, MtDMI2 and MtDMI3/MtCCaMK (the common symbiosis signaling module) and the transcriptional regulators MtNSP1, MtNSP2 and MtERN1 [42–44]. To investigate whether induction of *MtD27* expression by rhizobium LCOs is also dependent on this signaling module, we conducted qRT-PCR on LCO-susceptible root zones of these mutant plants, using *MtENOD11* as control. This revealed that *MtD27* expression is not induced in *Mtdmi1*, *Mtdmi2* and *Mtdmi3* mutant roots (Fig. 4b), indicating that symbiotic induction of *MtD27* requires the common signaling module genes. Previously, we demonstrated that *MtD27* expression in non-inoculated roots is dependent on MtNSP1 and MtNSP2 [17]. Here, we found that *MtD27* expression is still induced by LCOs in an *Mtnsp1*, *Mtnsp2* and *Mtnsp1Mtnsp2* mutant background, albeit significantly lower than in wild-type roots (~2-3-fold vs ~15-fold, respectively) (Fig. 4c). Next, we monitored *MtD27* expression in the *Mtern1* mutant, in which LCO-induced *MtENOD11* expression is blocked [45] (Fig. 4d). In this mutant, the induction of *MtD27* by rhizobium LCOs is about half of that in the wild type (Fig. 4e). Taken together, these results show that *MtD27* is a rhizobium LCO-responsive gene whose expression in a symbiotic context is largely dependent on the common signaling module and the transcriptional regulators MtNSP1, MtNSP2 and in part MtERN1.

Previously it was shown in *Vicia sativa* that the LCO inducibility of early nodulin genes is indirect, as an inhibition of protein synthesis by cycloheximide (CHX) blocks early nodulin genes expression [46]. As *MtD27* displays a comparable expression pattern as *MtENOD11*, we tested whether this gene is a primary target of rhizobium LCO signaling. To this end, *M. truncatula* seedlings were grown in Fåhraeus slides for 3 days and pre-treated with 50 μM CHX for 30 min prior to LCO treatment (10^–9^ M, 3 h). The expression of *MtD27* and *MtENOD11* in the susceptible root zone was monitored by qRT-PCR. This showed that in control plants *MtD27* expression is elevated by CHX treatment (Fig. 4f), suggesting that active protein synthesis is required to keep *MtD27* expression at basal levels. Additionally it showed that, like *MtENOD11*, *MtD27* expression is induced by rhizobium LCOs in the absence of CHX, but not in the presence of CHX (Fig. 4f). This indicates that induction of *MtD27* expression by rhizobium LCOs requires new protein synthesis and therefore suggests that induction of *MtD27* by rhizobium LCOs is indirect.

### *MtD27* is co-expressed with *MtCCD7* and *MtCCD8* during nodule formation

**Fig. 5:**
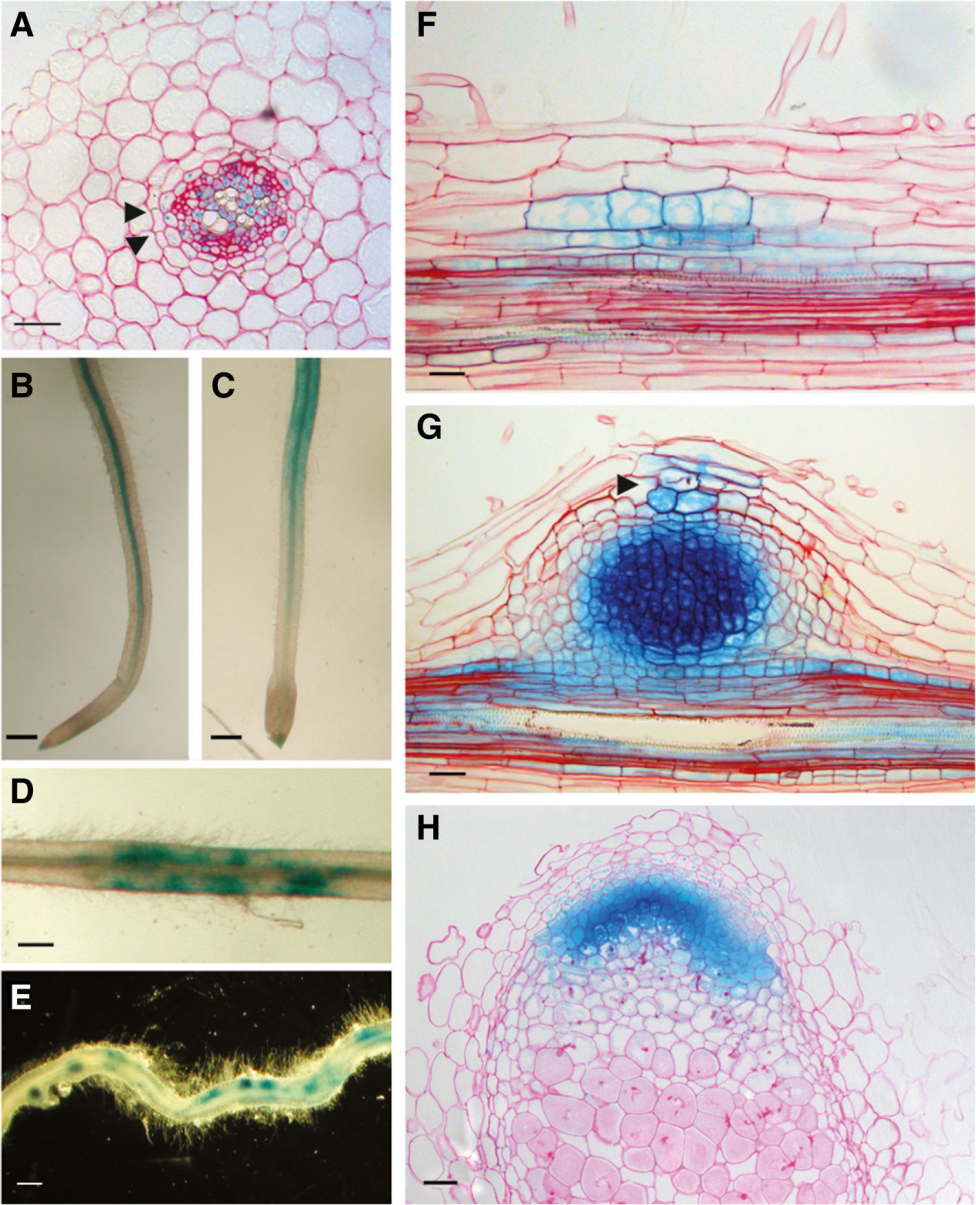
Spatial expression pattern of *MtD27* in *M. truncatula* roots and nodules. The *MtD27* spatial expression pattern was analyzed in *M. truncatula* transgenic roots expressing an *MtD27* promoter-reporter GUS construct. **a** Cross-section through a non-inoculated root. Arrowheads indicate casparian strips, which mark the endodermal cell layer. **b** Mock-treated control root. **c** Root treated with *S. meliloti* LCOs (10^-9^ M) for 3 h. **d** Root at four days post inoculation (dpi) with *S. meliloti* strain 2011. **e** Root at 7 dpi. **f** Longitudinal section through a root at 2 dpi. **g** Longitudinal section through a nodule primordium (7 dpi). The infection thread is indicated with an arrowhead. **h** Longitudinal section through an eighteen-day-old nodule. Scale bars are equal to 25 μm (**a**, **f** and **g**), 0.5 mm (**b-e**) and 50 μm (**h**). Sections were counterstained with Ruthenium Red

To obtain insight in the symbiotic function of *MtD27*, its spatiotemporal expression pattern is analyzed. For this, a ~1 kb fragment representing the 5’ region upstream of the translational start site was cloned into a binary transformation vector in front of a β-glucuronidase (GUS) encoding sequence. This construct was used to create *M. truncatula* compound plants carrying transgenic roots. In non-inoculated plants, grown on buffered nodulation medium (BNM) containing no nitrate, but a relatively high phosphate concentration (0.5 mM PO_4_^3-^), *MtD27* expression was observed in the vasculature and pericycle (Fig. 5a and b). LCO (~10^-9^ M) treatment for 3 h induced *MtD27* expression in the root epidermis (Fig. 5c).

Next, we determined the spatial expression of *MtD27* following inoculation with *S. meliloti* strain 2011. This showed that at 4 days post inoculation (dpi), the *pMtD27::GUS* transgenic roots showed a patched GUS staining, which was associated with rhizobium root hair infections (Fig. 5d). At 7 dpi, the expression in the root ceased, but GUS activity accumulated in nodule primordia (Fig. 5e). Sectioning nodule primordia revealed expression of *pMtD27::GUS* in dividing pericycle and cortical cells (Fig. 5f). This expression maintains in the developing nodule primordium (Fig. 5 g), but becomes more restricted in the mature nodule, where it is visible only in the meristem and distal infection zone (Fig. 5 h). Taken together, the LCO responsiveness and its spatial expression pattern in nodule primordia, nodule meristem and infection zone strongly support a symbiotic function of *MtD27*.

**Fig. 6:**
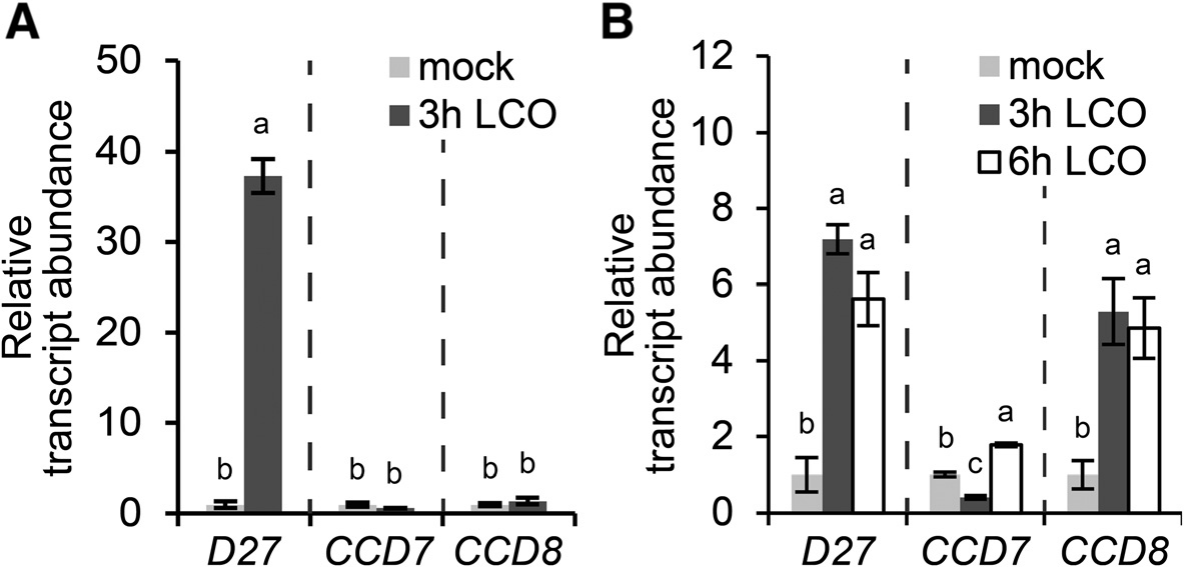
Expression of *MtD27*, *MtCCD7* and *MtCCD8* upon treatment with rhizobium LCOs. **a** Relative transcript abundance as determined by qRT-PCR of *MtD27*, *MtCCD7* and *MtCCD8* in *M. truncatula* root susceptible zones 3 h after mock or rhizobium LCO (10^-9^ M) treatment. RNA was isolated from plants grown in Fåhraeus slides. **b** Relative transcript abundance as determined by qRT-PCR of *MtD27*, *MtCCD7* and *MtCCD8* in *M. truncatula* root susceptible zones 3 or 6 h after mock or rhizobium LCO (10^-9^ M) treatment. RNA was isolated from plants grown on agar-solidified Fåhraeus medium supplemented with 1 μM AVG. Data shown represent means of 2-3 biological replicates that each were analyzed in 3-fold (technical replicates) ± SEM. For each gene, transcript abundance was normalized against that of the mock-treated wild type. Different letters above bars indicate statistical difference (*p* < 0.05, students’ *t*-test)

As strigolactone biosynthesis requires at least three enzymes, we tested whether besides *MtD27*, also *CCD7* and *CCD8* are responsive to rhizobium LCOs in *M. truncatula*. To this end, we first identified the putative orthologs of *CCD7* and *CCD8* from the *M. truncatula* genome based on homology to Arabidopsis genes. This revealed that one copy of *CCD7* (Medt7g045370; *MtCCD7*) and two copies of *CCD8* (Medtr3g109610 and Medtr7g063800) are encoded in the *M. truncatula* genome (Additional file 2). Medtr3g109610 was described previously as *MtCCD8* [6]. Analysis of the Medicago gene atlas showed that *MtCCD8* and Medtr7g063800 show a similar expression pattern, though expression of *MtCCD8* is about 10- to 20-fold higher when compared to Medtr7g063800 [39]. This also applies to expression of both genes during phosphate starvation and rhizobium LCO application (Additional file 3), and therefore we decided to focus on *MtCCD8* for the remainder of this study. Expression analysis by qRT-PCR on samples taken from plants grown in Fåhraeus slides showed that *MtCCD7* and *MtCCD8* were not induced at 3 h post rhizobium LCO application (Fig. 6a). In contrast, in roots grown on agar-solidified Fähraeus medium supplemented with aminoethoxyvinylglycine (AVG) expression of *MtCCD8* is induced ~5-fold 3 h after application of rhizobium LCOs, whereas *MtCCD7* expression was not substantially affected (Fig. 6b).

**Fig. 7:**
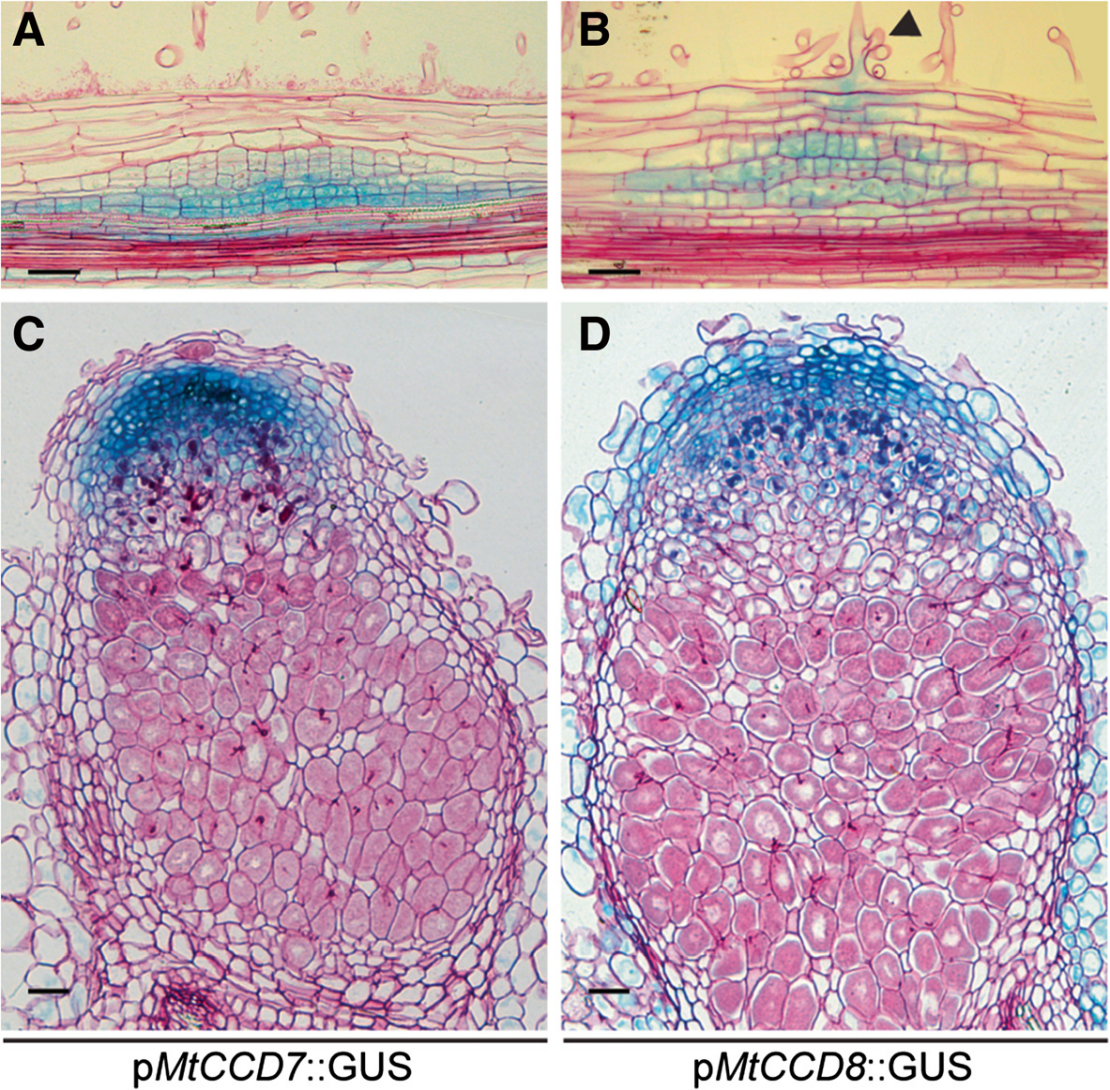
Spatial expression pattern of *MtCCD7* and *MtCCD8* in *M. truncatula* nodule primordia and mature nodules. Expression patterns were analyzed in *M. truncatula* transgenic roots expressing promoter-reporter GUS constructs. **a** Longitudinal section through a root expressing the *pMtCCD7::GUS* construct 2 days post inoculation (dpi) with *S. meliloti* strain 2011. **b** Longitudinal section through a root expressing the *pMtCCD8::GUS* construct at 2 dpi. Arrowhead points at an infection thread growing inside the root hair cell. **c** Longitudinal section through a mature nodule expressing the *pMtCCD7::GUS* construct. **d** Longitudinal section through a mature nodule expressing the *pMtCCD8::GUS* construct. Scale bars are equal to 50 μm. Sections were counterstained with Ruthenium Red

To determine the spatial-temporal expression pattern of *MtCCD7* and *MtCCD8* promoter-reporter GUS constructs were created and introduced in *M. truncatula* roots using *A. rhizogenes*-mediated transformation. We noted a basal expression pattern of both genes in the young root tip, including the susceptible zone and did not observe a discernible change in expression pattern of neither *MtCCD7* nor *MtCCD8* following LCO treatment (Additional file 4). This suggests that of the MtD27-MtCCD7-MtCCD8 biosynthesis module *MtD27* expression is most strictly controlled in a spatial-temporal manner. However, upon inoculation with *S. meliloti* it showed that in young (two-day-old) nodule primordia *MtCCD7* and *MtCCD8* are co-expressed with *MtD27* (Fig. 5f, 7a and b). Moreover, in mature nodules, *MtD27*, *MtCCD7* and *MtCCD8* are co-expressed in the nodule meristem and distal infection zone (Fig. 5 h, 7c and d).

Previous studies on strigolactone deficient mutants revealed that strigolactones at least in part contribute to root nodule formation and functioning [2–4]. To test whether *MtD27* function is required for rhizobium symbiosis, we determined the nodulation phenotype of *MtD27* RNAi roots. Under these conditions, the *MtD27* RNAi construct reduced *MtD27* expression by >90 % (Additional file 5a). Examination of the *MtD27* RNAi roots showed that they can be effectively nodulated (Additional file 5b). Sectioning ~40 nodules did not reveal any discernible difference in nodule morphology between nodules formed on control or *MtD27* RNAi roots (Additional file 5c and d). This suggests that either the reduction in *MtD27* expression is not sufficient to cause a phenotype or that *MtD27* is not essential for root nodule development and functioning.

### The induction of *MtD27* expression by phosphate deprivation is independent of the common symbiotic signaling cascade

**Fig. 8:**
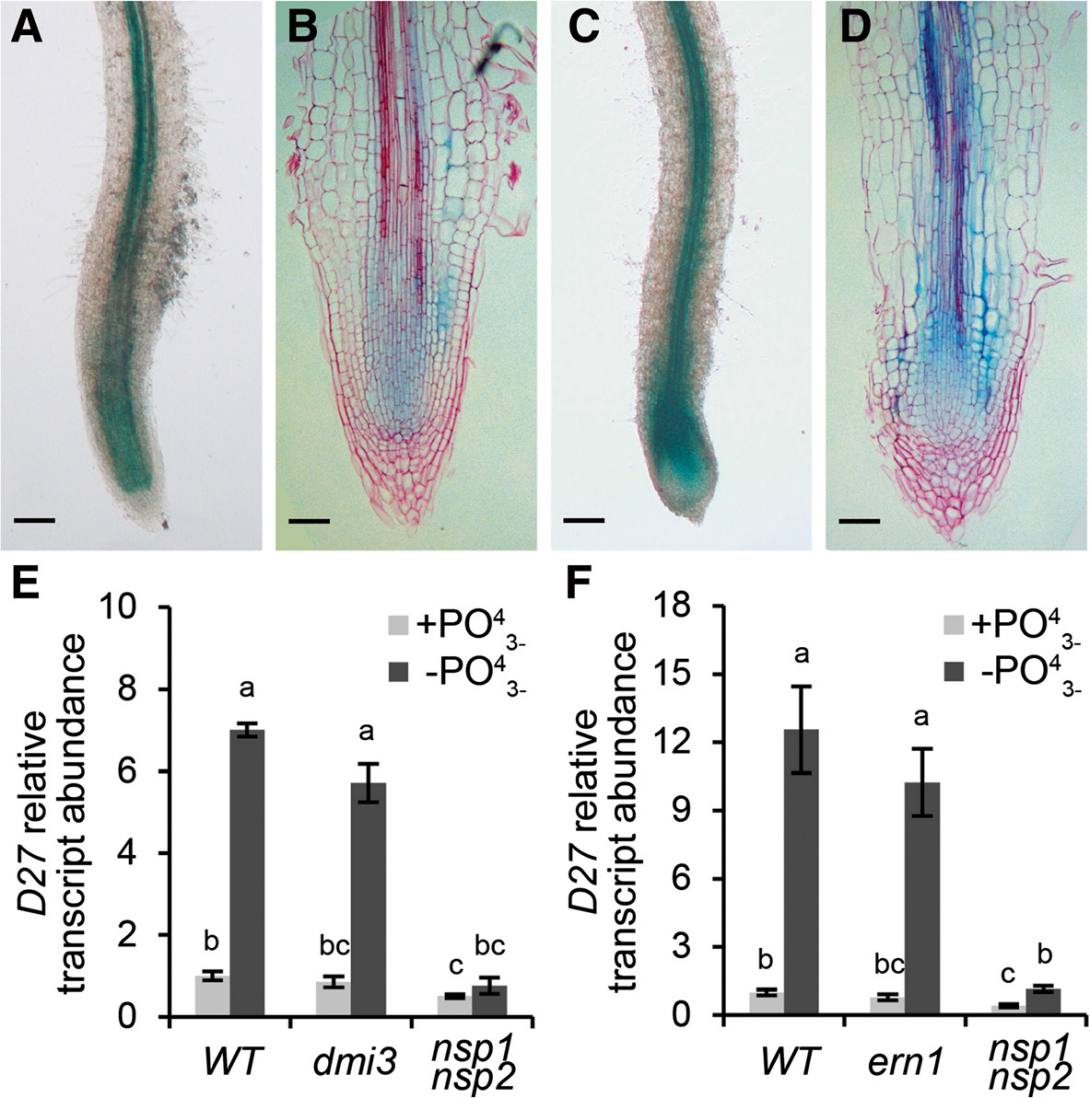
Spatial expression pattern of *MtD27* upon phosphate starvation. **a** Transgenic *M. truncatula* root expressing *pMtD27::GUS* grown in full nutrient condition (200 μM PO_4_^3-^). **b** Longitudinal section of the root shown in (**a**). **c** Expression of *pMtD27::GUS* in *M. truncatula* transgenic roots after 5 days of phosphate starvation (0 μM PO_4_^3-^). **d** Longitudinal section of the root shown in (**c**). **e** Relative transcript abundance as determined by qRT-PCR of *MtD27* in wild type (WT) and the *Mtdmi3* mutant and *Mtnsp1 Mtnsp2* double mutant after 2 days of phosphate starvation (0 μM PO_4_^3-^). **f** Relative transcript abundance as determined by qRT-PCR of *MtD27* in WT and the *Mtern1* mutant and *Mtnsp1 Mtnsp2* double mutant after 2 days of phosphate starvation. Scale bars are equal to 250 μm in (**a**) and (**c**) and 50 μm in (**b**) and (**d**). Sections were counterstained with Ruthenium Red. Data in (**e**,**f**) represent means of 3 biological replicates that each were analyzed in 3-fold (technical replicates) ± SEM. For each gene, transcript abundance was normalized against that of the mock-treated wild type. Different letters above bars indicate statistical difference (*p* < 0.05, students’ *t*-test)

Besides rhizobium LCOs, also phosphate starvation elevates *MtD27* expression in an NSP1 and NSP2-dependent manner [17]. Induction of *MtD27* by rhizobium LCOs requires MtDMI3/MtCCaMK and in part MtERN1. DMI3/CCaMK is positioned directly upstream of NSP1-NSP2 in the LCO signaling pathway [7, 47], whereas ERN1 functions in concert with NSP1-NSP2 to regulate expression of *MtENOD11* [48]. In line with this, we questioned whether MtDMI3/MtCCaMK and MtERN1 are also required to induce *MtD27* expression in response to a low phosphate status. First, we determined to what extent the spatial *MtD27* expression in *M. truncatula* roots is affected by different phosphate regimes. To this end, transgenic *M. truncatula* plants carrying the *MtD27* promoter-GUS reporter construct were grown in perlite for 2 weeks at high phosphate (200 μM PO_4_^3-^), and subsequently transferred to no phosphate (0 μM PO_4_^3-^) medium. Plant roots were stained histochemically for GUS activity 5 days after the transfer and compared to control roots. In all plants, GUS staining could be observed in the stele of the roots, as well as in the root apical meristems (Fig. 8a and b). Phosphate-starved roots displayed a much more intense staining than control roots, which was most clear in the root apical meristem (Fig. 8c and d). However, unlike treatment with rhizobium LCOs, phosphate starvation did not change the spatial expression pattern of *MtD27*.

Next, we determined whether MtDMI3/MtCCaMK and MtERN1 are also essential to mediate *MtD27* expression by phosphate starvation. To this end, *M. truncatula Mtdmi3* and *Mtern1* knockout mutants were grown in an aeroponic system containing medium with high phosphate (200 μM PO_4_^3-^) and subsequently transferred to the medium containing no phosphate for 2 days. To determine the expression of *MtD27*, qRT-PCR was conducted on RNA isolated from the bottom 2-3 cm of the root. This study reveals that in both mutants induction of *MtD27* in response to phosphate deprivation is similar to the induction found in roots of wild-type plants (Fig. 8e and f). This indicates that the phosphate response of *MtD27* expression is independent of the symbiotic signaling genes *MtDMI3/MtCCaMK* and *MtERN1*.

## Discussion

Several lines of evidence indicate that strigolactones play a role in the legume rhizobium symbiosis [1–6]. Here, we showed that expression of *MtD27*, a gene that is acting in the strigolactone biosynthesis pathway, is strongly elevated by rhizobium LCO-induced signaling. Additionally, we found that this gene is co-expressed with *MtCCD7* and *MtCCD8* in nodule primordia as well as in the infection zone of mature nodules. This suggests a putative function for these strigolactone biosynthesis genes during several stages of the legume-rhizobium interaction.

Studies with the strigolactone analog GR24 revealed a nodulation enhancing effect when applied exogenously [1, 5]. In line with this, a severe reduction in endogenous strigolactone levels due to mutations in *ccd7* or *ccd8* is linked to a moderate decrease in nodulation efficiency [2–4]. Foo and Davies [2] conclude that, although strigolactones influence nodule initiation they are not essential. We were unable to confirm these results in *MtD27* RNAi knockdown roots of *M. truncatula*. We cannot rule out that in our *MtD27* RNAi experiments *MtD27* expression is not sufficiently reduced to cause such moderate nodulation phenotype. Alternatively, phenotypes caused by an altered D27 function may be weaker to that of phenotypes of *ccd7* or *ccd8* mutants. This hypothesis finds support by studies in arabidopsis and rice, where shoot branching phenotypes were much more severe in *ccd7* and *ccd8* mutants when compared to *d27* [21, 38]. Based on this, Waters et al. [38] speculate on residual bioactive compounds present in *Atd27* mutants. If such a residual bioactive compound also exists in *M. truncatula*, it is possible that its activity is sufficient for proper nodule initiation and development.

It remains currently unknown how strigolactones promote nodule initiation. One possible mechanism is through promoting the formation of a nodular auxin maximum. Mathematical modelling predicts that such maximum is most likely created through a local reduction in the auxin transport capacity in the root cortex [49]. Such reductions in root auxin transport capacity have been observed following rhizobial inoculation [50, 51]. Strigolactone-deficient mutants of Arabidopsis show elevated auxin transport in both shoots and roots [52]. It is proposed that strigolactones act by targeting the PIN auxin-efflux carriers at both the gene expression and protein level [53–57]. Therefore, it is possible that rhizobium-induced strigolactone biosynthesis will affect auxin transport and as such contributes to create and/or maintain an auxin maximum during nodule formation. However, it is unlikely that strigolactones alone are sufficient to reduce the auxin transport capacity upon perception of rhizobium LCOs, as strigolactone-deficient mutants still form nodules, although less numerous than wild-type plants [2, 3]. Possibly, they could function redundantly to another signal, like for example cytokinin [51, 58–60] or flavonoids [61].

*MtD27* expression is elevated within 1-2 h post LCO application, by which it is among the earliest responsive genes. This transcriptional activation is under control of the rhizobium LCO signaling network, which includes MtDMI2, MtDMI1, MtDMI3/MtCCaMK, MtNSP1, MtNSP2 and in part MtERN1. We found that also expression of *MtCCD7* and *MtCCD8* was induced following application of rhizobium LCOs. However, these responses were less pronounced when compared to *MtD27.* Expression of *MtCCD7* was only slightly affected by application of rhizobium LCOs, whereas induction of *MtCCD8* was dependent on the growth system. Spatial-temporal expression analysis revealed that in a symbiotic context especially *MtD27* expression is strictly controlled in a spatial-temporal manner. Under non-symbiotic conditions *MtD27* is mainly expressed in the stele of the root, whereas rhizobium LCOs activate expression in the root epidermis. Such clear spatial-temporal regulation was not observed for *MtCCD7* nor *MtCCD8* as both genes have a much broader expression pattern under non-symbiotic conditions. Why *MtD27* is strictly controlled under symbiotic conditions remains unknown. Expression of *MtCCD7* and *MtCCD8* might not be rate limiting, or alternatively, induction of *MtD27* by rhizobium LCOs might be part of a priming response that prepares epidermal cells for the infection process [6].

Our data hint at a putative role for strigolactones in mature nodules, as *MtD27*, *MtCCD7* and *MtCCD8* are co-expressed in the nodule meristem and distal infection zone. This may suggest that strigolactones promote meristem functioning and/or rhizobial infection. Recently, it was shown that *MtD27* and *MtCCD8* are transcriptionally induced in infected root hairs [6]. Our data also indicate expression of *MtD27* and *MtCCD8* in cells that contain growing infection threads in the root nodule, supporting a putative function for *MtD27* and *MtCCD8* in the infection process. Furthermore, induction of *MtD27* expression by rhizobium LCOs is partly dependent on MtERN1, a transcription factor required for infection thread development [45].

**Fig. 9:**
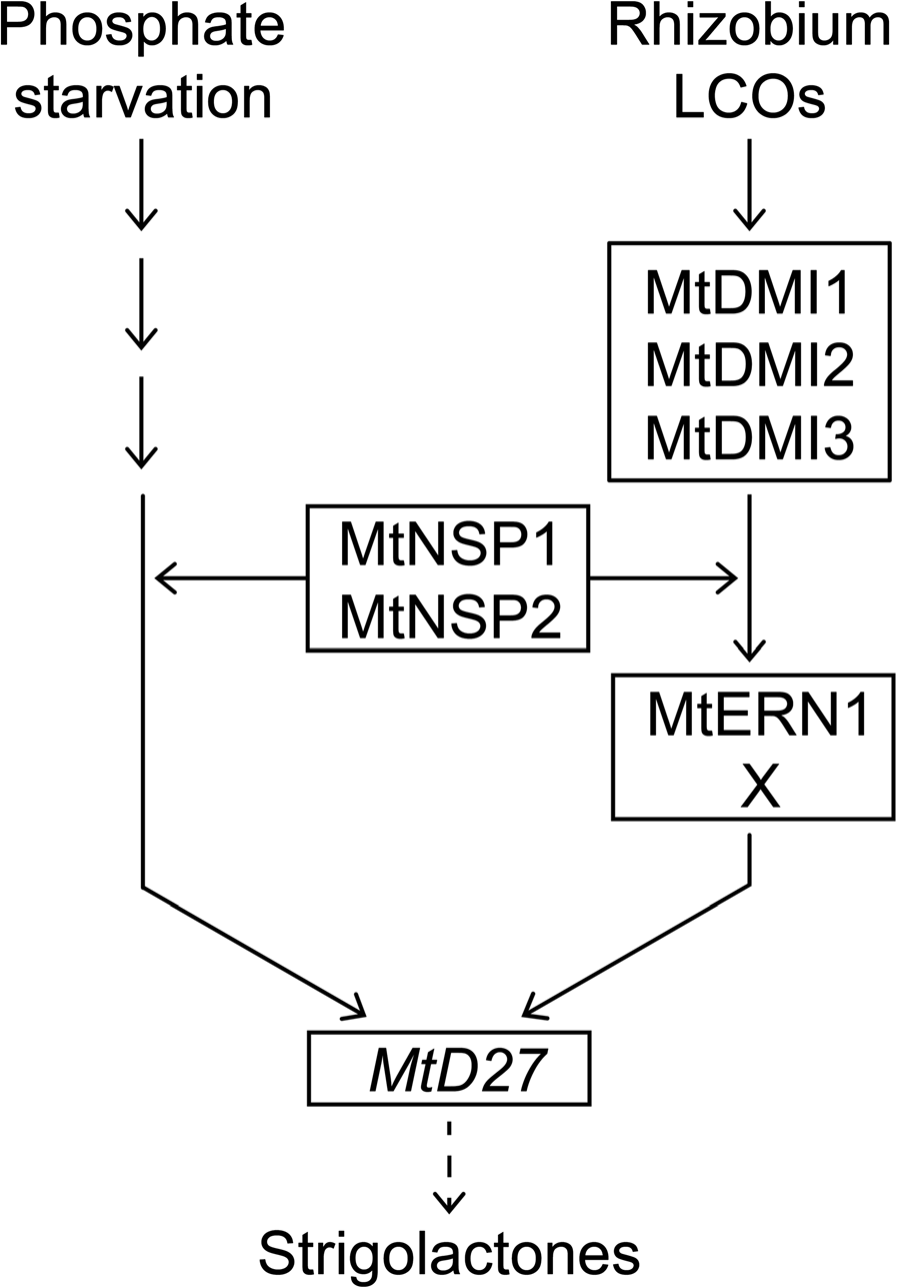
Schematic model depicting the induction of *MtD27* expression by phosphate starvation and rhizobium LCO-induced signaling. Rhizobium LCOs activate expression of *MtD27* through the common symbiosis signaling module consisting of MtDMI1, MtDMI2 and MtDMI3. Downstream of this module MtERN1 is required for full induction of *MtD27*. MtERN1 function is partly redundant, suggesting that MtERN1 might function in conjunction with or redundant to another unknown transcriptional regulator, indicated as X. This unknown factor might be MtERN2, a transcriptional regulator closely related to MtERN1 [48]. The pathway leading to activation of *MtD27* expression by phosphate starvation remains unknown. The GRAS proteins MtNSP1 and MtNSP2 are required for expression of *MtD27* during both phosphate starvation and following rhizobium LCO application. We propose that these proteins function in parallel to both signaling pathways, as previously already suggested for the LCO signaling cascade [7, 15]

*MtD27* is also transcriptionally activated by phosphate starvation stress and this induction is dependent on MtNSP1 and MtNSP2 [17]. We studied the spatial regulation of *MtD27* in response to phosphate starvation and found that the spatial expression pattern remains unchanged, but *MtD27* expression is increased in the stele and apical root meristem. The transcriptional activation of *MtD27* in response to the phosphate status in the environment coincides with an increased exudation of strigolactones [29, 30, 34]. Generally, it is anticipated that this response is contributing to the attraction of endomycorrhizal fungi, which enhance phosphate acquisition from the environment [33, 36]. We tested whether the induction of *MtD27* by phosphate starvation is dependent on the common signaling pathway as well as whether this response is (partially) dependent on MtERN1. Neither signaling components were involved in the phosphate starvation induced *MtD27* expression. This indicates that the signaling pathways regulating transcriptional activation of *MtD27* by rhizobium LCOs and phosphate starvation only share NSP1 and NSP2 (Fig. 9). Interestingly, *MtD27* transcript abundance is reduced in an *Mtnsp1Mtnsp2* mutant background when compared to wild type [17]. Furthermore, we found that induction of this gene upon phosphate stress or LCO signaling still occurs in these mutants, although at very moderate levels (Figs. 4e and 8f). Taken together, this supports the hypothesis that MtNSP1 and MtNSP2 function in a parallel pathway to facilitate induction of *MtD27* by rhizobium LCOs and phosphate starvation stress, rather than being part of the primary signaling cascades [7, 15] (Fig. 9).

The D27-CCD7-CCD8 biosynthetic module is largely conserved in higher plants. As we showed that *MtD27* is transcriptionally activated in a spatial-temporal manner in response to rhizobium LCOs in *M. truncatula* roots, this gene may represent an excellent marker gene to study rhizobium-induced signaling in a phylogenetic context.

## Conclusions

Here we showed that in *M. truncatula*, *MtD27* expression is rapidly increased upon perception of rhizobium LCOs. The gene remains expressed in the dividing cells of the nodule primordium and at subsequent stages its expression becomes confined to the nodule meristem and distal infection zone of the mature nodule. Analysis of the expression of *MtCCD7* and *MtCCD8* showed that they are co-expressed with *MtD27* in nodule primordia and mature nodules. Additionally, we show that symbiotic expression of *MtD27* as well as its expression during phosphate starvation is dependent on the GRAS-type regulators MtNSP1 and MtNSP2. This suggests that the NSP1-NSP2-D27 regulatory unit is co-opted to function in rhizobium symbiosis.

## Methods

### Plant materials and growth conditions

*M. truncatula* Jemalong A17, *dmi1-1* (C71) [9, 42], *dmi2-1* (TR25) [62], *dmi3-1* (TRV25) [10], *nsp1-1* (B85) [14, 42], *nsp2-2* (0-4) [13, 43], *nsp1 nsp2* [17] and *ern1* (*bit1-1*) [45] were used in this study. Plants were grown in a growth chamber at 20 °C under 16/8 photoperiod at 50 μmoles of photons m^-2^ s^-1^. For gene expression studies, plants were grown in modified Fåhraeus slides [40]. A single germinated seedling was placed in each slide, and medium was refreshed every 24 h. Seedlings were grown for 3 days, before subjecting them to a 3 h treatment with *Sinorhizobium meliloti* LCOs (~10^-9^ M), unless stated otherwise. Subsequently, 1 cm root segments were cut just above the root tip and snap-frozen in liquid nitrogen.

For analysis of transcriptional induction of *MtD27* by phosphate starvation, plants were grown as previously described [17]. This time, plants were grown for ~2.5 weeks before subjecting them to a two-day phosphate starvation regime. Subsequently, 2-3 cm root segments including the root tip were cut and snap-frozen in liquid nitrogen.

### Phylogenetic reconstruction

Predicted proteomes of *Glycine max* (Wm82.a2.v1) [63], *Lotus japonicus* (Lj2.5) [64], *Medicago truncatula* (Mt4.0v1) [65], *Oryza sativa* (v7.0) [66], *Populus trichocarpa* (v3.0) [67] and *Vitis vinifera* (Genoscope.12X) [68] were obtained through Phytozome 10 (http://phytozome.jgi.doe.gov/). These proteomes were searched by BLAST using *A. thaliana* proteins (TAIR10, www.arabidopsis.org) as query. For phylogenetic reconstruction, full length (predicted) protein sequences were aligned using MAFFT v7.017 [69] implemented in Geneious R6 (Biomatters, Auckland, New Zealand), using default parameter settings. After manual inspection, alignments were used for tree building using MrBayes 3.2.2 [70] implemented in Geneious R6, using default parameter settings, with the exception of the rate matrix, for which wag was used. Midpoint rooting was performed for better tree visualization.

### Vectors and constructs

For promoter-GUS reporter assays, a ~1 kb (*MtD27*) or ~2 kb (*MtCCD7* and *MtCCD8*) fragment upstream of the translational start site was amplified from *M. truncatula* Jemalong A17 genomic DNA using the primers listed in Additional file 6. The p*MtD27* fragment and a β-glucuronidase (GUS)-encoding sequence were recombined into a pDONR-L4L1 and pDONR-L1L2, thereby creating pENTR4-1_p*MtD27* and pENRT1-2_GUS, respectively. These two constructs were combined with a pENTR2-3_t35S and subsequently recombined into the binary destination vector pKGW-RR-MGW by a multisite gateway reaction (Invitrogen, Carlsbad, USA). The putative promoter fragments of *MtCCD7* and *MtCCD8* were recombined into a pENTR-D-TOPO vector (Invitrogen, Carlsbad, USA), creating pENTR1-2_p*MtCCD7* and pENTR1-2_p*MtCCD8*, respectively. Subsequently, both constructs were recombined into pKGWFS7-RR, containing a GUS-GFP fusion reporter, by a single-site gateway reaction (Invitrogen, Carlsbad, USA), creating pKGWFS7-RR_p*MtCCD7*-GUS and pKGWFS7-RR_p*MtCCD8*-GUS, respectively.

For RNAi-mediated knockdown of *MtD27*, a 268-bp fragment was amplified from *M. truncatula* Jemalong A17 root cDNA, using primer pairs MtD27i-F and MtD27i-R (see Additional file 6), and cloned into pENTR-D-TOPO (Invitrogen, Carlsbad, USA). The *MtD27* RNAi fragment was recombined into the DsRed-modified gateway vector pK7GWIWG2(II)-RR driven by the CaMV35S promoter [71] to obtain the binary construct pK7GWIWG2(II)-RR-p35S-MtD27-RNAi. For the empty vector control, a pENTR containing a ~70 bp multiple cloning site was recombined into pK7GWIWG2(II)-RR to obtain the binary plasmid pK7GWIWG2(II)-RR-p35S-RNAi-control.

All vectors used in this study contain p*AtUBQ10::DsRED1* as selection marker [72]. All cloning vectors are available upon request from Plant Systems Biology (V.I.B.-Ghent University).

### Plant transformation and treatments

*A. rhizogenes*-mediated root transformation of *M. truncatula* was performed as previously described [72]. For treatments with rhizobium LCOs, compound plants were transferred to agar-solidified buffered nodulation medium (BNM; 0.9 % Daishin agar (Duchefa, Haarlem, The Netherlands)) [73] containing 1 μM aminoethoxyvinylglycine (AVG) (Sigma, St. Louis, USA). After 3 days, ~100 μl of rhizobium LCOs (~10^-9^ M) was pipetted on top of the root susceptible zone. After 3 h, roots were fixed in 90 % acetone and subsequently stained for GUS activity. For nodulation assays, compound plants were transferred to perlite and watered with Fåhraeus [74] medium without nitrate. One week after transfer, plants were inoculated with *S. meliloti* strain 2011 (OD_600_ = 0.05-0.1). For the phosphate starvation experiment, compound plants were transferred into perlite and watered with half-strength Hoagland medium [75]. After one week, plants were removed from perlite and washed three times with demineralized water to get rid of the nutrient salts. Plants were re-planted in fresh perlite and watered with half-strength Hoagland medium with (200 μM PO_4_^3-^) or without (0 μM PO_4_^3-^) phosphate, respectively. After 5 days, plants were removed from perlite and stained for GUS activity.

### Histochemical staining and microtome sectioning

For histochemical GUS staining of *M. truncatula* roots and nodules, samples were first rinsed three times with 100 mM phosphate buffer (PBS; pH = 7.2). Samples were transferred to GUS-staining buffer (contains 2 mM K_3_Fe(CN)_6_, 2 mM K_4_Fe(CN)_6_, 10 mM EDTA, 0.1 % Triton X-100 and 1 mg/ml X-Gluc salt (Duchefa, Haarlem, The Netherlands) in 100 mM PBS, pH = 7.2) and placed under vacuum for 30 min. Next, the samples were incubated in the dark at 37 °C for 3 h. Stained roots were rinsed with PBS (pH = 7.2) three times to stop the reaction.

For historesin embedding, roots and nodule samples were fixed with 5 % glutaraldehyde PBS (pH = 7.2) solution overnight. After fixation, the samples were rinsed with PBS (pH = 7.2) three times and dehydrated through ethanol gradients (20 %, 40 %, 60 %, 80 % and 100 %). Afterwards, the samples were embedded in Technovit 7100 (Heraeus-Kulzer, Wehrheim, Germany), according to the manufacturer’s protocol. GUS-stained samples were sectioned to 7 μm using a microtome (Reichert-Jung, Leica Microsystems, Rijswijk, The Netherlands) and stained with 0.1 % Ruthenium Red for 15 min. Images were taken using a Leica DM5500B microscope equipped with a Leica DFC425C camera (Leica Microsystems, Wetzlar, Germany). Images were digitally processed using Photoshop CS6 (Adobe Systems, San Jose, USA).

### qRT-PCR analysis

RNA was isolated from snap-frozen root material using the plant RNA kit (E.Z.N.A. Omega Biotek, Norcross, USA) following the supplier’s manual. cDNA was synthesized from 1 μg total RNA using the iScript cDNA synthesis kit (Bio-Rad, Hercules, USA). qRT-PCR reactions were set up in a 20 μl reaction system with 2× iQ SYBR Green Super-mix (Bio-Rad, Hercules, USA) and the iQ5 Real-time PCR detecting system according to the manufacturer’s manuals. All primers used in this study were designed using the qPCR settings of Primer3Plus [76]. Relative expression values were calculated using the 2^-ΔΔCt^ method, using *M. truncatula* ubiquitin (*MtUBQ10*) and polypyrimidine tract-binding protein (*MtPTB*) as reference genes. Statistical significance was determined based on students’ *t*-test (unpaired, two tailed, equal variance). All primers used in this study are listed in Additional file 6.

### Strigolactone analysis

*M. truncatula* root exudates and root extracts were purified and concentrated as previously described [17, 30] with minor modifications. Compound (*MtD27* RNAi and Empty vector control) plants were grown on perlite and watered twice a week with 50 mL half-strength MS medium (Duchefa, Haarlem, The Netherlands). Seven days prior to strigolactone analysis pots were washed with 3 volumes half-strength MS medium without PO_4_^3-^ (Duchefa, Haarlem, The Netherlands) to initiate phosphate starvation. Strigolactone quantification was performed by comparing retention time and mass transitions with those of an available didehydro-orobanchol standard using ultra-performance LC coupled to MS/MS using [^2^H_6_]2’-epi-5-deoxystrigol as an internal standard, as previously described [77]. Didehydro-orobanchol MS/MS fragmentation spectra of *M. truncatula* were obtained as previously described [17]. Results were subjected to students’ *t*-test (unpaired, two tailed, equal variance).

### Medicago gene atlas IDs

Probe IDs used for analysis of gene expression using the Medicago gene expression atlas [39] are listed in Additional file 7.

### Availability of supporting data

All relevant supporting data can be found within the supplementary files accompanying to this article. The phylogenetic trees were deposited in TreeBase (www.treebase.org) and can be accessed using the following link: (http://purl.org/phylo/treebase/phylows/study/TB2:S18414).

## Additional files

Additional file 1:
**Relative transcript abundance of** ***MtD27*** **and Medtr7g095920 upon phosphate starvation.** Relative transcript abundance of *MtD27* and Medtr7g095920 in roots of plants grown under low (20 μM) or high (2 mM) phosphate conditions. Data were obtained from the Medicago gene atlas [39]. Data represent means of three replicates ± SEM. Transcript abundance was normalized against *MtD27* transcript abundance in roots grown under high (2 mM) phosphate conditions. Different letters above bars indicate statistical difference (*p* < 0.05, students’ *t*-test). (TIFF 737 kb) Additional file 2:
**Bayesian phylogeny of CCD1, CCD7 and CCD8 proteins.** Bayesian phylogeny of CCD1, CCD7 and CCD8 proteins from Arabidopsis (At), soybean (*Glycine max*) (Glyma), *M. truncatula* (Medtr), rice (Os), petunia (*Petunia hybrida*) (Ph), poplar (*Populus trichocarpa*) (Potri), pea (*Pisum sativum*) (Ps) and grapevine (*Vitis vinifera*) (VIT). CCD1 proteins were included as an outgroup. Medicago proteins are highlighted in red. Branch support is indicated by posterior probabilities. Terminals are labeled by their gene or genbank identifier. Mid-point rooting was applied for better tree visualization. (TIFF 3321 kb) Additional file 3:
**Relative transcript abundance of** ***MtD27*** **,** ***MtCCD7*** **,** ***MtCCD8*** **and Medtr7g063800 during phosphate starvation and upon rhizobium LCO treatment.** (a) Transcript abundance of *MtD27*, *MtCCD7*, *MtCCD8* and Medtr7g063800 in roots of plants grown under low (20 μM) or high (2 mM) phosphate conditions. (b) Transcript abundance of *MtD27*, *MtCCD7*, *MtCCD8* and Medtr7g063800 in mock-treated roots (-LCO) or roots treated with rhizobium LCOs (+LCO) for 6 or 24 h. Data were obtained from the Medicago gene atlas [39]. Data represent means of three replicates ± SEM. Transcript abundance was normalized against 0*MtD27* transcript abundance in roots grown under high (2 mM) phosphate conditions (a) or in the 6 h mock-treated sample (b). Different letters above bars indicate statistical difference (*p* < 0.05, students’ *t*-test). (TIFF 2450 kb) Additional file 4:
**Spatial expression pattern of** ***MtCCD7*** **and** ***MtCCD8*** **upon application of rhizobium LCOs.** *MtCCD7* and *MtCCD8* spatial expression patterns were analyzed in *M. truncatula* transgenic roots expressing promoter-reporter GUS constructs. Roots were mock-treated or treated with *S. meliloti* LCOs (10^-9^ M) for 3 h. Scale bars are equal to 0.5 mm. (TIFF 1705 kb) Additional file 5:
**Nodule phenotype after knock-down of** ***MtD27*** **through RNAi.** (a) Relative transcript abundance as determined by qRT-PCR of *MtD27* in *M. truncatula* transgenic roots expressing an empty vector control construct (EVi) or *MtD27* RNAi construct (D27i). (b) Number of nodules formed on plants bearing transgenic roots harboring an empty vector control construct (EVi) or *MtD27* RNAi construct (D27i). (c) Section through a nodule formed on a root expressing the empty vector control construct. (d) Section through a nodule formed on a root expressing the *MtD27* RNAi construct. Scale bars are equal to 100 μm. Data shown in (a,b) represent means of 6 (a) or 5 (b) biological replicates ± SEM. Different letters above bars indicate statistical difference (*p* < 0.05, students’ *t*-test). (TIFF 4222 kb) Additional file 6:
**Primer sequences of all primers used in this study.** The recombination sites in the sequences are shown in italic. (CSV 1 kb) Additional file 7:
**Probe IDs used in this study as in the Medicago gene atlas.** (CSV 180 bytes)

## Abbreviations

BNM: Buffered nodulation medium
CHX: Cycloheximide
Dpi: Days post inoculation
GUS: β-glucuronidase
LCO: Lipo-chitooligosaccharide
PBS: Sodium phosphate buffer
qRT-PCR: Quantitative reverse transcriptase polymerase chain reaction
