# Supplementary figures and images for: The strigolactone biosynthesis gene DWARF27 is co-opted in rhizobium symbiosis
Source: BMC Plant Biol. 2015 Oct 26;15:260. doi: 10.1186/s12870-015-0651-x (PMC4624177; doi:10.1186/s12870-015-0651-x)

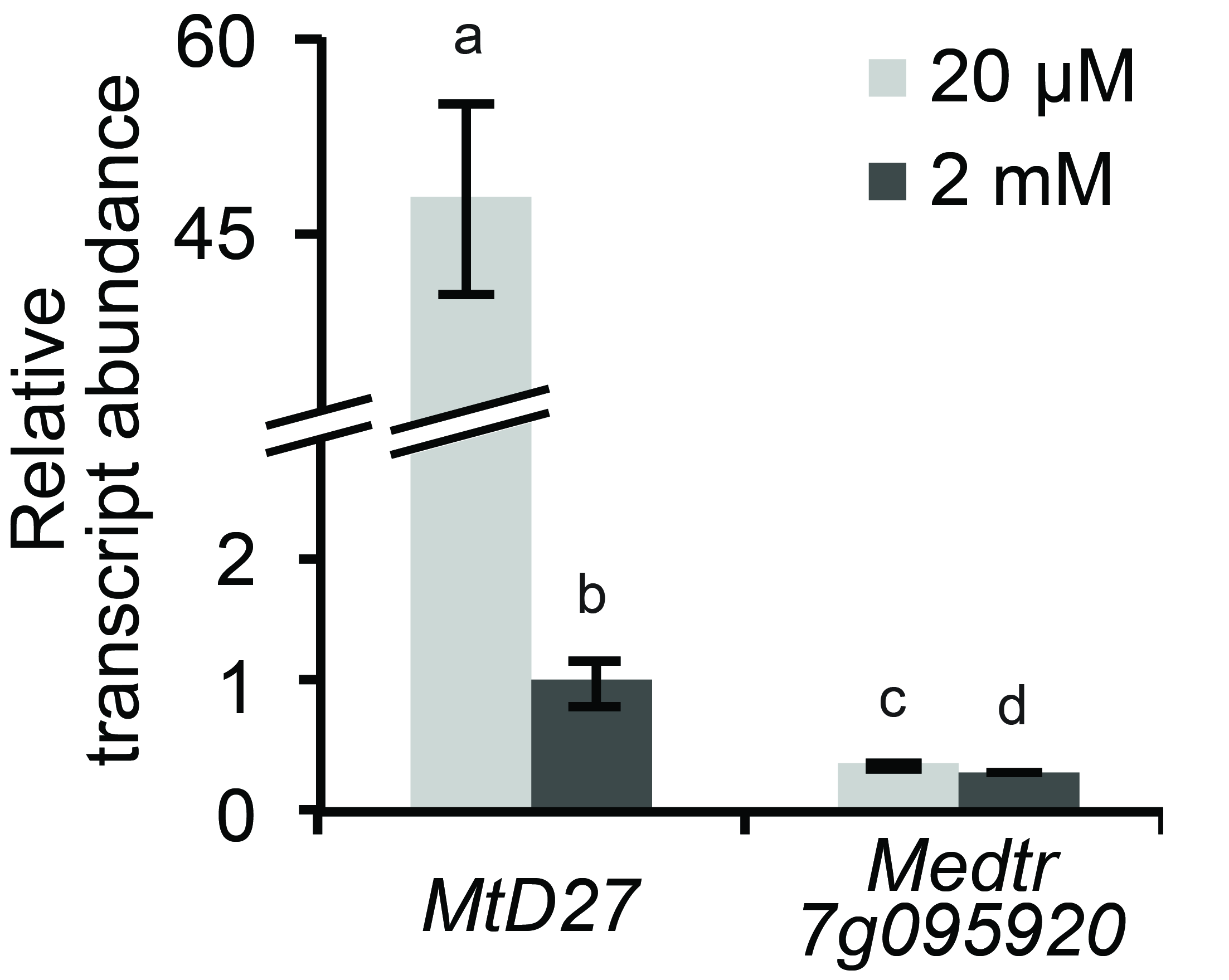

Supplement: Additional file 1: — Relative transcript abundance of MtD27 and Medtr7g095920 upon phosphate starvation. Relative transcript abundance of MtD27 and Medtr7g095920 in roots of plants grown under low (20 μM) or high (2 mM) phosphate conditions. Data were obtained from the Medicago gene atlas [39]. Data represent means of three replicates ± SEM. Transcript abundance was normalized against MtD27 transcript abundance in roots grown under high (2 mM) phosphate conditions. Different letters above bars indicate statistical difference (p < 0.05, students’ t-test). (TIFF 737 kb) [file 12870_2015_651_MOESM1_ESM.tif]

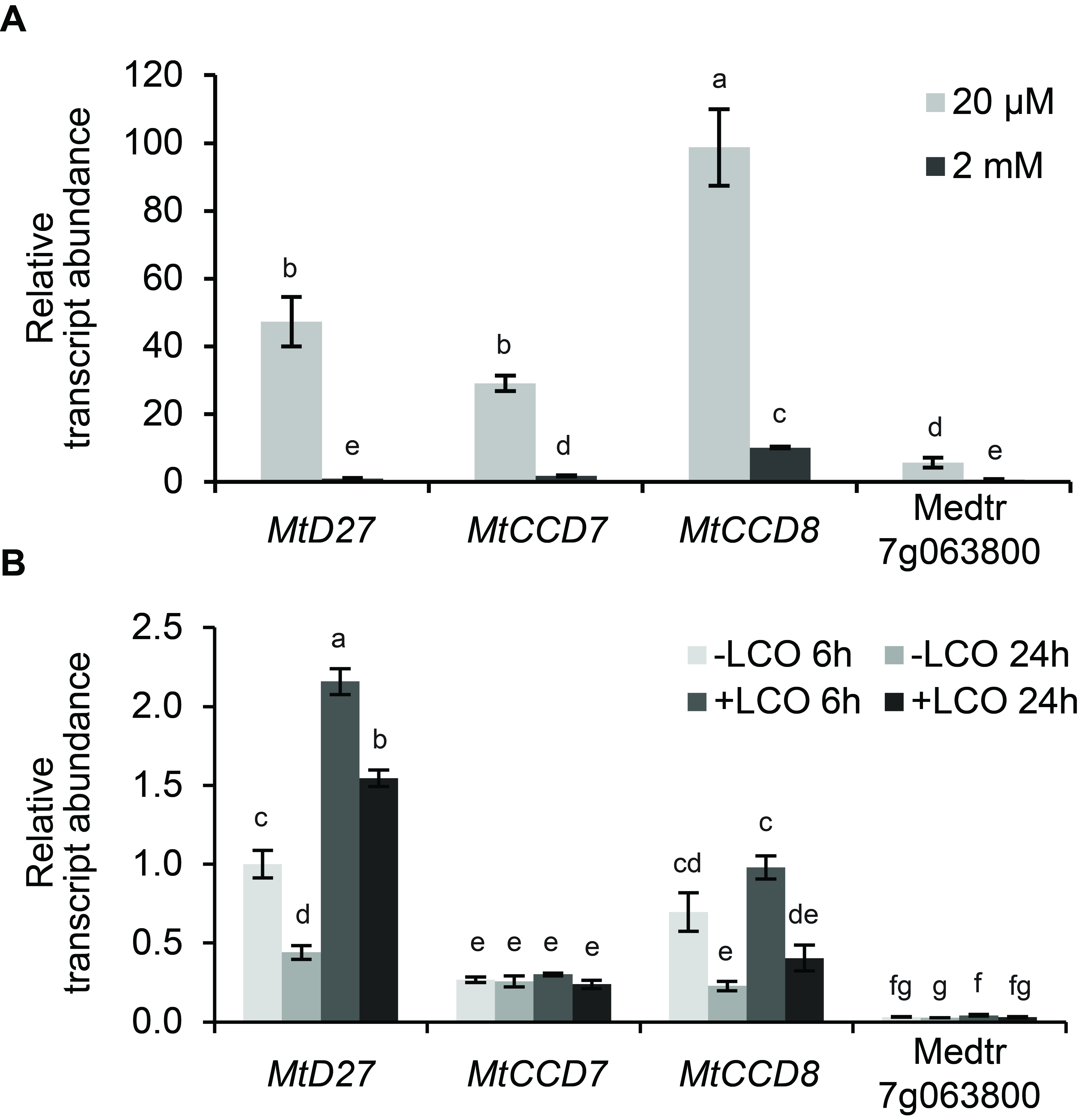

Supplement: Additional file 3: — Relative transcript abundance of MtD27 , MtCCD7 , MtCCD8 and Medtr7g063800 during phosphate starvation and upon rhizobium LCO treatment. (a) Transcript abundance of MtD27, MtCCD7, MtCCD8 and Medtr7g063800 in roots of plants grown under low (20 μM) or high (2 mM) phosphate conditions. (b) Transcript abundance of MtD27, MtCCD7, MtCCD8 and Medtr7g063800 in mock-treated roots (-LCO) or roots treated with rhizobium LCOs (+LCO) for 6 or 24 h. Data were obtained from the Medicago gene atlas [39]. Data represent means of three replicates ± SEM. Transcript abundance was normalized against 0MtD27 transcript abundance in roots grown under high (2 mM) phosphate conditions (a) or in the 6 h mock-treated sample (b). Different letters above bars indicate statistical difference (p < 0.05, students’ t-test). (TIFF 2450 kb) [file 12870_2015_651_MOESM3_ESM.tif]

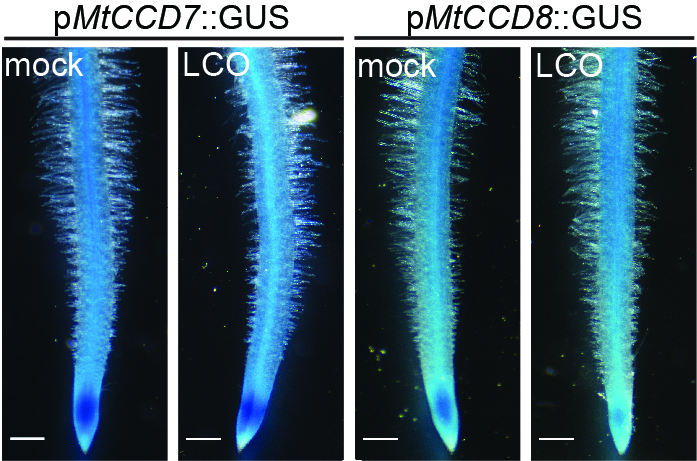

Supplement: Additional file 4: — Spatial expression pattern of MtCCD7 and MtCCD8 upon application of rhizobium LCOs. MtCCD7 and MtCCD8 spatial expression patterns were analyzed in M. truncatula transgenic roots expressing promoter-reporter GUS constructs. Roots were mock-treated or treated with S. meliloti LCOs (10-9 M) for 3 h. Scale bars are equal to 0.5 mm. (TIFF 1705 kb) [file 12870_2015_651_MOESM4_ESM.tif]

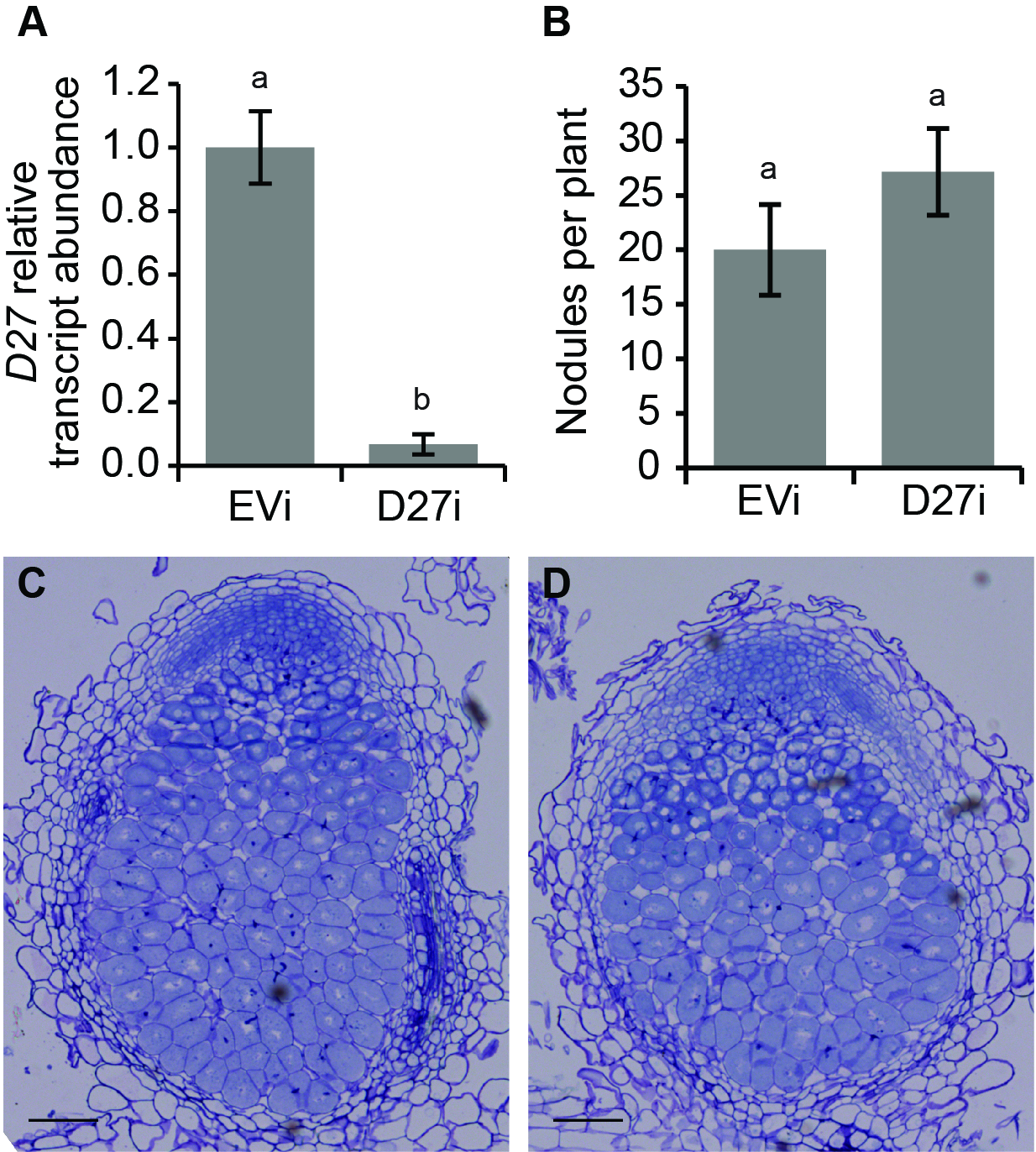

Supplement: Additional file 5: — Nodule phenotype after knock-down of MtD27 through RNAi. (a) Relative transcript abundance as determined by qRT-PCR of MtD27 in M. truncatula transgenic roots expressing an empty vector control construct (EVi) or MtD27 RNAi construct (D27i). (b) Number of nodules formed on plants bearing transgenic roots harboring an empty vector control construct (EVi) or MtD27 RNAi construct (D27i). (c) Section through a nodule formed on a root expressing the empty vector control construct. (d) Section through a nodule formed on a root expressing the MtD27 RNAi construct. Scale bars are equal to 100 μm. Data shown in (a,b) represent means of 6 (a) or 5 (b) biological replicates ± SEM. Different letters above bars indicate statistical difference (p < 0.05, students’ t-test). (TIFF 4222 kb) [file 12870_2015_651_MOESM5_ESM.tif]
